# Studying epigenetic complexes and their inhibitors with the proteomics toolbox

**DOI:** 10.1186/s13148-016-0244-z

**Published:** 2016-07-18

**Authors:** David Weigt, Carsten Hopf, Guillaume Médard

**Affiliations:** Chair of Proteomics and Bioanalytics, Technical University of Munich, Emil Erlenmeyer Forum 5, 85354 Freising, Germany; Center for Applied Research in Biomedical Mass Spectrometry (ABIMAS), Mannheim University of Applied Sciences, Paul-Wittsack-Str. 10, 68163 Mannheim, Germany; HBIGS International Graduate School of Molecular and Cellular Biology, Heidelberg University, Im Neuenheimer Feld 501, 69120 Heidelberg, Germany

**Keywords:** Target deconvolution, Complex identification, Affinity matrix, Photo-cross-linking, Affinity purification, Immunoaffinity purification

## Abstract

Some epigenetic modifier proteins have become validated clinical targets. With a few small molecule inhibitors already approved by national health administrations and many more in the pharmaceutical industry pipelines, there is a need for technologies that can promote full comprehension of the molecular action of these drugs. Proteomics, with its relatively unbiased nature, can contribute to a thorough understanding of the complexity of the megadalton complexes, which write, read and erase the histone code, and it can help study the on-target and off-target effect of the drugs designed to modulate their action. This review on the one hand gathers the published affinity probes able to decipher small molecule targets and off-targets in a close-to-native environment. These are small molecule analogues of epigenetic drugs conceived as protein target enrichment tools after they have engaged them in cells or lysates. Such probes, which have been designed for deacetylases, bromodomains, demethylases, and methyltransferases not only enrich their direct protein targets but also their stable interactors, which can be identified by mass spectrometry. Hence, they constitute a tool to study the epigenetic complexes together with other techniques also reviewed here: immunoaffinity purification with antibodies against native protein complex constituents or epitope tags, affinity matrices designed to bind recombinantly tagged protein, and enrichment of the complexes using histone tail peptides as baits. We expect that this toolbox will be adopted by more and more researchers willing to harness the spectacular advances in mass spectrometry to the epigenetic field.

## Background

Proteomics has proven to be a reliable ally to study drug-protein interactions and protein-protein interactions for the last 20 years [1]. Substantial progress in the mass spectrometry techniques now allows to measure accurately and quantitatively the proteins contained in complex samples. Advances in the technology allow shorter measurement times while digging deeper in the proteomes. Hydrolases and kinases have been the great beneficiaries of past efforts to characterize sub-proteomes and their inhibitors [2, 3]. But they are not the only ones. The field of epigenetic proteomics could develop further as it accompanies the thriving phase of epigenetic drug discovery endeavors and successes [4–6] outlined throughout this special issue.

## Chemical proteomics methods to identify epigenetic drug targets and off-targets

Among the contributions made by proteomics to the field of epigenetics [5], chemical proteomics [7] has emerged as a solid methodology to decipher small molecule targets and off-targets via so-called compound pulldowns. These consist of the enrichment of the sub-proteome that is bound by a small molecule in a complex lysate or even directly in cells and generally mass spectrometry readout. Because of unspecific binding or cross-linking, the sub-proteome enriched by inactive molecules can be compared as a control. However, a better strategy is to compete with the free small molecule, preferably in a dose-dependent fashion, where the fit of the curves will help determine the reality of the newly found targets. Additionally, such a competition assay will give EC_50_s, which can be converted to *K*_d_ values if a pulldown of the drug vehicle control pulldown flow-through is performed to assess the depletion of each protein [8].

It has to be noted that the foremost advantage of the chemical proteomics approach is that the assay can be performed in disease-relevant systems, where the proteins are close to the native state, contain the relevant PTMs, and are engulfed in their complexes.

The ability of chemical proteomics approaches to characterize a comprehensive spectrum of targets and off-targets in cells or tissues is of fundamental importance for a thorough understanding of compound promiscuity or polypharmacology [9] in these systems, i.e., the bothersome or welcome fact that a single drug often engages multiple targets, some of which are unknown during its discovery. Chemical proteomics therefore serves as a key link between diverse drug responses on a cellular or organismal level and a compound’s selectivity that is being increasingly scrutinized in the emerging field of systems pharmacology [10]—in epigenetics or beyond.

For example, Maleszewska et al. observed that, at low concentration, the HDAC2 inhibitor valproic acid increased H4 deacetylation in C6 glioma cells. This contradictory effect was hypothesized to be due to various known off-targets of valproic acid [11]. Moreover, the compound LY294002, which has been used as a PI3K inhibitor in more than 5000 PubMed-referenced publications, has clear PI3K-independent effects in cells. A chemical proteomics study recently revealed that the so far incomprehensible effects of this tool inhibitor (shared by a PI3K-inactive structural analogue) most likely result from activity against bromodomain proteins [12].

This may serve as an example for a widely accepted chemical proteomics strategy to distinguish members of the targeted protein complex from mistakenly captured off-targets, namely the use of orthogonal probes. Due to their chemically different structures, orthogonal probes are expected to not only share specificity for the targeted protein complex but also have different sets of off-targets [13]. Vice versa, this approach can also be a valuable tool to purposely screen for off-targets of known inhibitors to elucidate mechanisms behind complicated phenotypic effects.

The term activity-based protein profiling (ABPP) has been generalized to any kind of pulldowns featuring some covalent bond between the small molecule of interest and the proteins it is bound to. This wording was meaningful when it was coined for the study of hydrolases: the probe was effectively hijacking the chemical mechanism of the enzymatic reaction, since the molecules were designed to react analogous to a substrate. It is however questionable if a binding followed by proximity driven photo-cross-linking can be assimilated to activity. Indeed, it carries with it the confusion of activity assay versus binding assay. As recently in the kinase field, activity profiling was claimed to be achieved with affinity matrices made out of type I kinase inhibitors before this conclusion was recently proven to be a seldom case, it appears important to be somewhat careful with this shift of meaning [14]. We are therefore choosing to avoid the ABPP acronym in the review, when the molecules described are not hijacking the mechanism of the proteins. Most of the probes are binding in the active pocket of the enzymes without being analogues of reaction intermediates and are therefore affinity probes. Some strategies make full use of a strong affinity that allow the maintenance of the interaction long-enough so that the interactors can be identified after washing. Other strategies feature a subsequent step of cross-linking, which bears the advantage of covalent linkage and the possibility of locating the binding event. These strategies, however, also give rise to unspecific cross-linking where the kinetics play a crucial role.

**Fig. 1:**
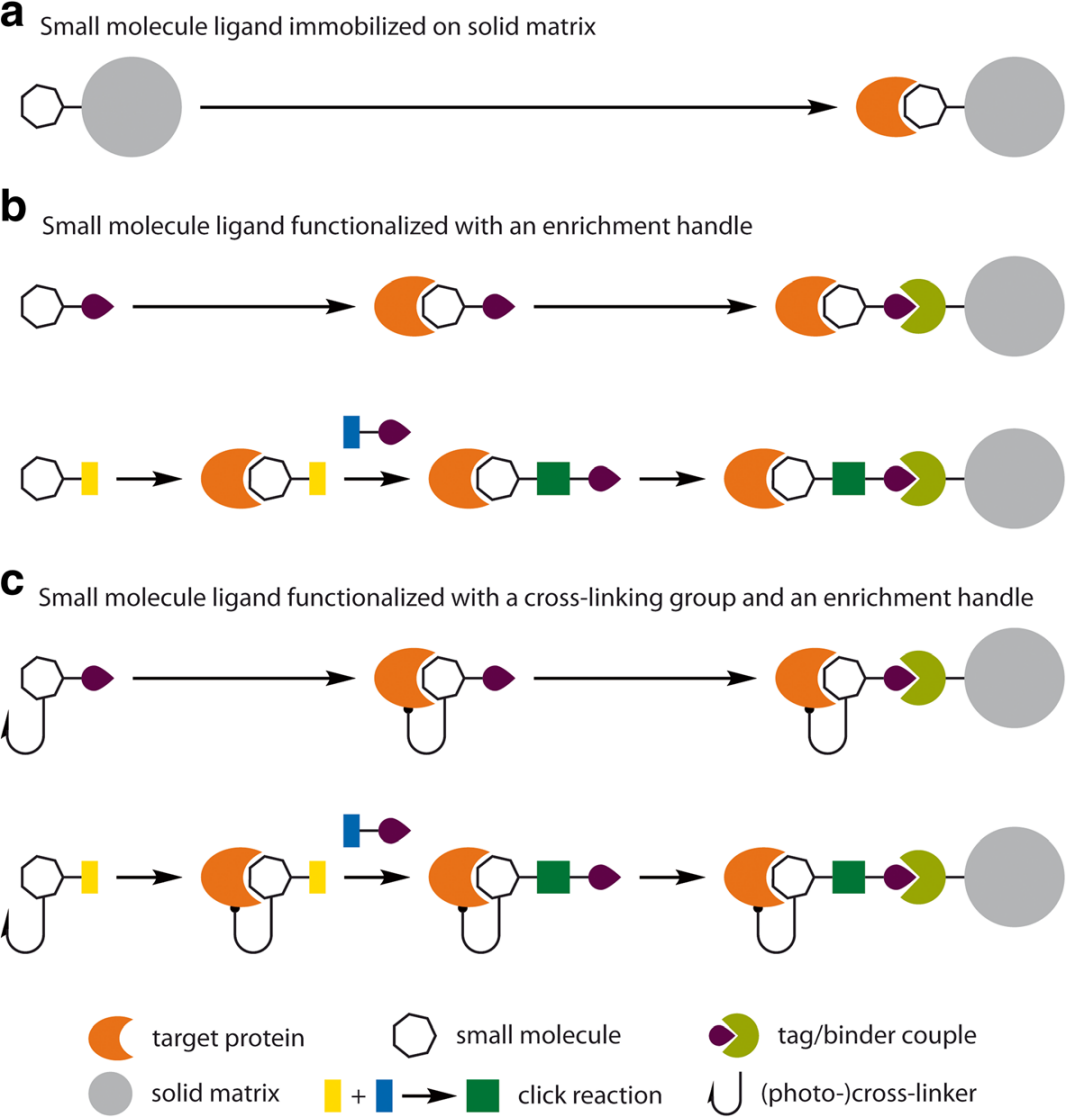
Affinity probes for the identification of drug targets by chemical proteomics strategies. An analogue of the small molecule is synthesized that **a** is covalently attached to a solid matrix or **b** possesses an enrichment handle or **c** possesses a cross-linking moiety and an enrichment handle

The conceptually simplest approach to decipher the targets of a drug is to immobilize a linkable analogue of the molecule on a solid matrix and to proceed to the enrichment of the binders out of a cell or tissue lysate. This approach is incompatible with the *in cellulo* binding that is addressable by a molecule equipped with a handle allowing post-lysis pulldowns. In this case, a cross-linker can also be added. It has to be noted that any modification of the initial molecule can impair binding, that the bulk and length of the linker matters, and that cross-linking can be relatively low-yielding and unspecific [15]. Hence, we propose to distinguish (Fig. 1) between the:

- Small molecule ligand immobilized on a solid matrix. Different solid matrices can be envisioned, the most common being Sepharose beads or magnetic beads.
- Small molecule ligand functionalized with an enrichment handle. This enrichment handle can be a biotin moiety, allowing subsequent enrichment with a streptavidin matrix. It can also be a biorthogonal tag allowing for further enrichment using click reactions [16, 17].
- Small molecule ligand functionalized with a cross-linking group and an enrichment handle. The cross-linking group are very often photoreactive functionalities such as benzophenones, aryl azides, or diazirines [18, 19].

### Deacetylase enrichment probes

Since the HDAC inhibitor Vorinostat (aka SAHA) has been the epigenetic drug the most studied by chemical proteomics, we shall begin by describing the various reported approaches using linkable analogues of this molecule. They constitute a good overview of what is possible in the field: all the approaches described above (Fig. 1) have indeed been successfully used for the identification of Vorinostat targets.

A team of researchers in Cellzome immobilized a linkable analogue of Vorinostat (p-aminomethyl Vorinostat) and an analogue of Givinostat on Sepharose beads to obtain an affinity matrix able to enrich HDAC1, 2, 3, 6, 8, and 10 out of K562 cell extracts. They then set the free Vorinostat and 15 other HDAC inhibitors (PCI-34051, MC-1293, valproic acid, PCI-24781, Romidepsin, Tacedinaline, Entinostat, BML-210, Mocetinostat, Scriptaid, Belinostat, Apicidin, Panobinostat, Dacinostat, Trichostatin A) to compete for binding with the beads using six different drug concentrations. The proteins eluted from the beads were labeled with TMT and measured simultaneously to obtain the dose-response curves in one mass spectrometry measurement. Kd_app_s for all the drug-native protein interactions could hence be obtained, establishing the selectivity profiles of these inhibitors. The authors then adapted the chemical proteomics method to high-throughput replacing mass spectrometry readout by multiplexed fluorescent antibody on “dot blot” arrays. This allowed them to profile a small library of molecules in the lysate of Jurkat and Ramos cells for HDAC1, 2, 3, and 6 binding. Bufexamac, an anti-inflammatory drug with unknown target, was identified as preferentially inhibiting HDAC6 in this screen, and its profile was further assessed by the original chemical proteomics profiling assay revealing its selectivity for HDAC6 and 10 in the 10 μM range [20]. The immobilized Vorinostat was later also used to profile dual HDAC/BET inhibitor binding to class I and IIb HDACs [21]. Lu et al. also immobilized Vorinostat using a p-amino Vorinostat analogue and enriched binders out of Hela cell lines which they compared to the proteins enriched by control beads with a mass spectrometry readout [22]. Salisbury and Cravatt introduced in 2007 an analogue (SAHA-BPyne) of Vorinostat featuring a benzophenone moiety for UV induced cross-linking and a terminal alkyne that was used to click on azido-rhodamine for gel visualization or onto an azide containing biotin analogue for enrichment with avidin beads. HDAC1, 2, 3, and 6 could be identified as targets after on-bead digestion and mass spectrometry readout in some cancer cell line lysates. The probe could also be used *in cellulo*. No other direct binder of Vorinostat was identified by the method [23]. Others created their capture tool based on Vorinostat and featuring a biotin tag and an aryltrifluoromethyldiazirine moiety attached by extended hydrophilic linkers. They identified HDAC1, 2, 3, and 6 as well as 13 other proteins as specifically enriched by the probe out of HepG2 cell lysate, among which they confirmed ISOC2 as a direct binder [24]. Petukhov and coworkers also created a Vorinostat derivative with a 3-azido-5-azidomethylbenzyloxy moiety appended to the 4-position of the Vorinostat phenyl, allowing UV cross-linking with the 3-azido group and enrichment by click chemistry with the 5-azidomethyl group and biotin-alkyne/avidin agarose. However, this tool was not used to enrich targets out of complex biological samples but to identify binding poses of Vorinostat in HDAC8 by studying the cross-linked peptides [25]. Another photo-cross-linking Vorinostat analogue was designed by Luo and coworkers featuring a benzotetrazole equipped with a terminal alkyne handle. Upon irradiation, the probe could cross-link the carboxylic acid moiety of a HDAC1 and HDAC2 glutamic acid residue adjacent to the binding site of the drug in HepG2 cell lysate and live cells. The alkyne handle was used for further click reaction for visualization (rhodamine-azide) or enrichment with streptavidin beads (biotin-azide) before western blot readout. The modification site was identified by mass spectrometry using purified proteins, preventing the identification of off-targets [26]. A recent approach used the chloroalkane capture tag as an enrichment handle which can be efficiently and very quickly bound to the commercial Promega HaloTag magnetic particles [27]. The Promega authors compared Vorinostat-choloroalkane to Vorinostat-biotin for in cellulo target engagement and found the first to have a 2.3-fold reduced potency compared to untagged Vorinostat while the second had a 16-fold reduced potency, an effect which was due to biotin interference with the binding, as proven by a BRET assay. This difference translated in the targets identified by pulldowns followed by elution with free Vorinostat, where the Vorinostat-chloroalkane HaloTag strategy allowed to show binding to HDAC1, 2, 3, 6, 8, and 10 whereas the Vorinostat-biotin strategy only allowed to identify HDAC6. The *in cellulo* target engagement of Vorinostat-chloroalkane and kinetics of the HaloTag capture proved to be excellent and thus avoided the use of cross-linking moieties to stabilize the interactions. In addition to these targets, the thiol dioxygenase ADO and the serine/threonine phosphatase CPPED1 could be identified as targets. The authors confirmed that Vorinostat is a direct inhibitor of these two metallo enzyme by BRET competition assay. Also the conversion of cysteamine to hypotaurine was inhibited when purified ADO was incubated with Vorinostat, possibly shedding light on the reported efficacy of Vorinostat in Huntington’s disease.

Trapoxin with its epoxyketone moiety has been shown to be an irreversible inhibitor. This should have hampered the use of a simple pulldown and elution strategy with a solid matrix functionalized with a Trapoxin analogue. The first designed strategy was, therefore, featuring a disulfide containing linker that would have been cleaved in the elution step. However, this approach was reported as unsuccessful whereas a non-cleavable linker allowed to elute HDAC1. The authors had, therefore, to conclude that this inhibitor is a covalent but reversible inhibitor [28, 29]. This matrix is the only one reported here that qualifies as ABPP since the epoxide is believed to react in mimicry to the acetyl carried by the lysine the enzyme is removing. Already in this seminal work performed two decades ago, which led to the identification of mammalian HDAC1 by Edman degradation, the immobilized Trapoxin matrix was competed not only with Trapoxin but also with Trichostatin, allowing to establish that these two inhibitors of histone deacetylation have a common binding site (or that, at least, the binding of one impairs the binding of the other one by structural rearrangement of the proteins).

Salisbury and Cravatt took their SAHA-BPyne further and in another publication compared it to other HDAC probes featuring benzophenone and terminal alkynes attached in various positions to different HDAC binders or potential binders. These included probes inspired by CI99. However, they found their initial SAHA-BPyne to be the most interesting tool [30]. The Gottesfeld group reported that two of their pimelic diphenylamid series featuring an anilinine zinc binding group were amenable to proteomics experiments. They were complemented by a BPyne moiety for the purpose and used in the biological context of Friedreich’s ataxia. Whereas HDAC3 is the preferred target over HDAC1 and 2, the authors could only evidence its presence by western blot while HDAC1 and 2 were clearly identified in the mass spectrometry readout [31, 32]. Chen and coworkers have reported the synthesis of a probe featuring a BPyne moiety as a tool to decipher the targets of cinnamic hydroxamic acids in the frame of an anti-hepatitis C virus agent drug discovery program. Since they report that the parent para-trifluoromethyl cinnamic hydroxamic acid has submicromolar activity for HDAC6 and 8, it is likely that the probe will also enrich these HDACs and maybe others in their future work [33]. Albrow and coworkers created a series of photoaffinity probes based on Pandacostat, TFMK, and TMP269, featuring a benzophenone photo-cross-linker and either a biotin or an alkyne enrichment handle. Whereas no derivative of TMP269 could retain the HDAC4 potency of the parent compound, one TFMK-BPyne analogue retained potency and selectivity to remain a class IIa selective molecule and, impressively, one Pandacostat-BPyne proved to be even superior to Pandacostat in its pan-HDAC potency against recombinant HDACs. Using HeLa lysates, however, comparison of the enriched proteins with the Pandacostat-BP-biotin immobilized on streptavidin-coated agarose with and without competing Pandacostat-BPyne could only identify HDAC 1, 2, 3, 6, and 8 as targets, failing to achieve the elusive class IIa enrichment and underscoring the difficulty to translate results obtained with truncated versions of the class IIa HDACs [34].

Among histone deacetylases, Sirtuins (also known as class III HDACs) are quite distinct since unlike all other HDACs which use Zn^2+^ as a cofactor, they need NAD^+^ to act as deacetylating enzymes and play their part in epigenetic regulation [35]. Hence, other HDACs inhibitors cannot promiscuously bind to the Sirtuin class, and dedicated capture tools have to be designed in order to profile their inhibitors. Sauve and coworkers designed an ABPP strategy using a *N*-thioacetyl-lysine peptide which, under catalysis of the sirtuin, displaces the nicotinamide of an analogue of NAD^+^ to form a stalled thioimidate. They designed the NAD^+^ analogue to contain an aminooxy biorthogonal tag (linker positioned at the 6-position of the adenine) which could be reacted with an aldehyde moiety tethered to the biotin enrichment handle further used to enrich the covalently attached proteins with streptavidin functionalized agarose beads [36]. Jung and coworkers designed a biotin functionalized Sirt2 selective inhibitor which exploits an isotype specific pocket arising from a major rearrangement of the active site upon inhibitor binding. Pulling down with magnetic streptavidin beads, they proved the selectivity of enrichment of Sirt2 over Sirt1 out of a native HL60 cell culture using western blot as a readout [37]. Dose-dependent competition with free drugs and mass-spec readout is still to come in order to evaluate by chemical proteomics the possible off-targets of their new class of inhibitors.

### Bromodomain probes

Using HepG2 hepatocyte cell line containing an apolipoprotein A1 luciferase reporter, researchers at GSK identified a benzodiazepine able to potently induce this gene. In order to deconvolute the target of the new class of compounds they had developed, a linkable analogue of the best molecule (I-BET762; I-BET; GSK525762) was synthesized. They compared the proteins retained by an agarose matrix functionalized with this linkable analogue (I-BET721; N-I-BET; GSK923121) to the proteins retained on a control matrix with the inactive enantiomer (GSK525768). Addition of the free drug would elute the retained proteins which were identified by mass spectrometry readout to be BRD2, BRD3, and BRD4. The matrix was further used to show that the drug interacts with the bromodomain containing N-terminal (1–473) truncate of BRD2 and not the C-terminal truncate (473–801) [38]. With this matrix in hand, GSK has since complemented their characterization of new BET inhibitors by chemical proteomics assays. An I-BET762 dose-response competitive assay in HL60 cell lysate allowed to generate EC_50_ values for the three inhibited BET proteins [39]. Immunobloting allowed to visualize that the new analogue I-BET151 was also preventing BRD4 to be enriched by the beads in HL60, MV4;11, and RS4;11 cell extracts [39]. I-BET295, DUAL946, and two other BET inhibitors (all 10 μM) were used to compete for binding with the matrix in HL60 extracts showing the binding to BRD2, 3, and 4. DUAL946 and another candidate were then profiled in a dose-response manner to obtain Kd_app_s [21].

Yao’s group prepared analogues of the benzodiazepine GW841819X functionalized with a diazirine photo-cross-linker and three different enrichment handles (an alkyne moiety and two cyclopropenes). After evaluation of the probes, two of them were used for in situ labeling of HepG2 cells with and without JQ1 as a competitor. The labeled proteins were clicked with azido-biotin or tetrazin-biotin before enrichment with avidin agarose beads. Comparing the overlaps and the background obtained with negative control probes, they established a list of 48 higher-confidence potential targets of JQ1. Among these, they confirmed by western blot that DDB1 and RAD23B were not enriched when JQ1 was co-incubated with the probes [40].

Of note is the synthesis of a biotinylated version of JQ1 to investigate the genome-wide binding of this bromodomain inhibitor to the BET bromodomain family members BRD2, BRD3, and BRD4 in MM1.S multiple myeloma cells (Chem-seq). After binding of the biotinylated JQ1 to its targets in cells or lysates, the chromatin-associated proteins were cross-linked to DNA with formaldehyde, which allowed to enrich with streptavidin beads the DNA fragments bound to the drug-target complex. Subsequent sequencing of these DNA fragments established the binding *loci* of the drug target [41].

Researchers in Pfizer discovered tropolone methyl ether derivatives to be CREBBP and BRD4 submicromolar binders. Interestingly, this acetyl lysine mimicking moiety is an inherent photo-cross-linker and the authors utilized this property: they appended an alkyne to their lead structure and after incubation with a BRD4 spiked K562 cell lysate under UV irradiaton, they could click and enrich BRD4 as proven by western blot. JQ1 was proven to compete to a certain extent. Endogenous BRD4 could, however, not be enriched, probably due to the fact that tropolones also cross-link tubulin [42].

Bromodomain binders can also be found among kinase inhibitors. A Cellzome team immobilized the commonly used PI3K probe LY294002 as well as LY303511, a PI3K inactive compound acting similarly in a number of studies independently of PIK3 pathway. Comparison of the proteins of HL60 cell extracts enriched by the two matrices evidenced a large overlap with PI3Ks, PRKDC, mTOR, and CK2 as clear exceptions. Notably, BRD2, BRD3, and BRD4 were the most abundant proteins when performing pulldowns with both the matrices. Unique targets of LY294002 such as the PI3Ks could be confirmed by dose response competitive assays of LY294002 and LY303511 against the immobilized LY294002 matrix. All three BETs were found to be inhibited similarly by both molecules. Corrected by the depletion factor, the apparent binding constants were around 1 μM, only two to four times more than the constants for the PI3Ks, and can possibly explain the PI3K independent effects observed [12]. To decipher if the drug is binding to the bromodomains or to a previously suggested kinase domain, the authors set up dose-response experiments with MgATP and I-BET151, a previously developed BET inhibitor, in competition with the immobilized LY294002 matrix. ATP did not prevent the matrix to bind BRD4, whereas the known BET inhibitor did, in a dose-dependent manner with an IC_50_ of 160 nM as evidenced by immunoblotting, establishing bromodomains as the binding site. A surprise arose when the inhibitor was used in a competition assay against the I-BET121 matrix described earlier or against the immobilized drug: the found IC_50_s readout by western blot was 40 times higher when the I-BET121 matrix was used but was similar to the one found when an immobilized H4K5acK8acK12ac matrix was used. This was consistent with LY294002 being selective for only one of the two bromodomains of the BETs while I-BET121 is not. To confirm this hypothesis, the team used yet another chemical proteomics approach which they combined with cellular biology: they compared the binding of the immobilized inhibitors to flag-tagged BRD4 isoform C point mutants overexpressed in HEK293T cells. Whereas the I-BET121 matrix could enrich wild-type Y97A and Y390A but not Y97/390A proteins, neither the LY303511 nor the N-LY294002 matrix could enrich the Y97A mutant but still enriched the wild-type and Y390A mutants. As Y97A affects the first bromodomain and Y390A the second bromodomain, this constitutes a piece of evidence that LY294002/LY303511 acts as a true histone H4 mimetic only binding to the first bromodomain of BETs [12].

In the light of this work and two independent reports of several X-ray structures of kinase inhibitors bound to bromodomains [43, 44], we here wish to report that many matrices designed to enrich kinases also enrich bromodomain containing proteins. Analyzing the raw data generated by Kuster and coworkers to characterize such matrices [8, 14, 45–48], it appeared that Cpd2, linkable SB203580, VI16743, and to a lesser extent Bisindolylmaleimide X, CTx0294885 and linkable Dasatinib are able to enrich BRD2, 3, and 4. Interestingly, the JAK enriching matrix 3b [48] could enrich ATAD2 in all three replicates. Further characterization will be necessary to define their binding modes and usability in competition experiments.

We have noted how compounds have been profiled with the repertoire of bromodomains enriched by an affinity matrix made out of a linkable molecule other than the one to study. This evidently creates blind spots and the results cannot be seen as a full profile. To alleviate the need to synthesize at least one linkable analogue for every drug (the presence of the linker can also create blind spots), akin to what has been done for kinases with Kinobeads [8, 49] or MIBs [50], a combination of affinity probes has been developed to profile in a more unbiased way potential bromodomain inhibitors and gain insight in their selectivity. Named “bromospheres” such a sepharose-based matrix enriching 19 bromodomain containing proteins has been used to profile three new ATAD2 inhibitors in HuT-78 mixed chromatin and nuclear extracts. After TMT labeling and mass spectrometry readout, dose-response curves of the molecules for the endogenous proteins were obtained. However, the structures of the three molecules forming this matrix have yet to be revealed [51].

### Demethylase probes

The discoverers of GSK-J1 validated the target engagement and selectivity of the drug using a chemical proteomics approach. They created a linkable analogue (GSK-J3) which they immobilized on Sepharose beads. This matrix was able to capture Flag-tagged JMJD3 and Flag-tagged UTX from transiently transfected HEK-293 cells and could efficiently be competed with 100 mM GSK-J1. HL-60 cells were treated with phorbol myristate acetate to trigger the expression of JMJD3, which could be enriched specifically by the matrix. Competition with 100 mM GSK-J1 produced the disappearance of the western blot band for endogenous JMJD3, while mass spectrometry readout concluded to the exquisite selectivity of the drug [52]. Bush et al. created a set of tripodal (pharmacophore, cross-linking moiety, and enrichment handle) IOX1 (5-carboxy-8-hydroxyquinoline) derivatives by Ugi four-component reaction and evaluated their efficiency in cross-linking isolated EGLN1 [53]. Since IOX1 is a broad spectrum 2-oxoglutarate oxygenase inhibitor with submicromolar activity against a range of histone demethylases [54], these probes could serve in competition assays to assess the selectivity of new inhibitors. Pushing further the idea of immobilizing unspecific 2-oxoglutarate-dependent dioxygenase binders, Joberty et al. created five affinity probes that they immobilized on Sepharose beads. Two of these molecules were oxoglutarate analogues, another probe was based on a known bipyridyl scaffold while the two others were nicotinic acid derivatives that the authors identified in a screen. With the obtained matrices, they could enrich 40 different dioxygenase enzymes from human cells out of the around 60 known such proteins, as identified by mass spectrometry. The discovery of such matrices allowed for the study of natural cofactor oxoglutarate and oncometabolites binding across the panel of enriched enzymes (notably 18 Jumonji-type proteins) by competitive pulldown assays/mass spectrometry readout. Moreover, with such a technological platform in place, profiling of small molecules inhibitors were performed. The Jumonji demethylase inhibitors JHDM-I1, GSK-J1, a pyrido [3,4-*d*]pyrimidin-4(3*H*)-one derivative, a 3-amino-pyridine-4-carboxylate derivative and a 3-cyano-pyrazolo-pyrimidinone derivative were profiled and were shown to exhibit varying potencies and selectivities for this family of proteins [55]. Hence, the chemical proteomics approach with mixed affinity matrix was proven to be a suitable tool to study the affinity and selectivity of existing and future demethylase inhibitors.

### Methyltransferase probes

Jin and coworkers reported the synthesis of a biotin derivatized analogue of UNC0638, an inhibitor of the lysine methyltransferase G9a, which could be used to coat streptavidin magnetic beads. This affinity matrix was then used to enrich the target protein out of HEK293T whole cell extracts. Binding to G9a could be inhibited by competition with the free drug as proven by western blot analysis [56]. In another report the authors disclose the immobilization on Sepharose beads of the aminopropyl analogue of UNC0638. This affinity matrix was used in combination with the SILAC technique (stable isotope labeling by amino acids in cell culture) to determine the differential abundance of more than 2000 proteins enriched from nuclear extracts of BMDMs under different inflammatory states [57]. Based on a panel selectivity study for UNC0638 [58], they considered that the probe only bound G9a as a substrate competitive ligand and used the obtained list of proteins as potential G9a interactors. These two affinity matrices should be useable to generate dose response binding curves in competition experiments, first to confirm the excellent selectivity of UNC0638 and then to also validate the complexes.

Indeed in chemical proteomics pulldowns, many targets are enriched together with the complexes they are engulfed in. When performing competition experiments with a dilution series of a drug, typical dose-response curves of the direct targets of the drug are to be observed and also matching curves for the proteins which are co-enriched as member of stable complexes. This feature can be put to fruition to study epigenetic complexes as will be detailed in the next chapter.

## Beyond the study of the isolated modulators: tools to study complexes

**Fig. 2:**
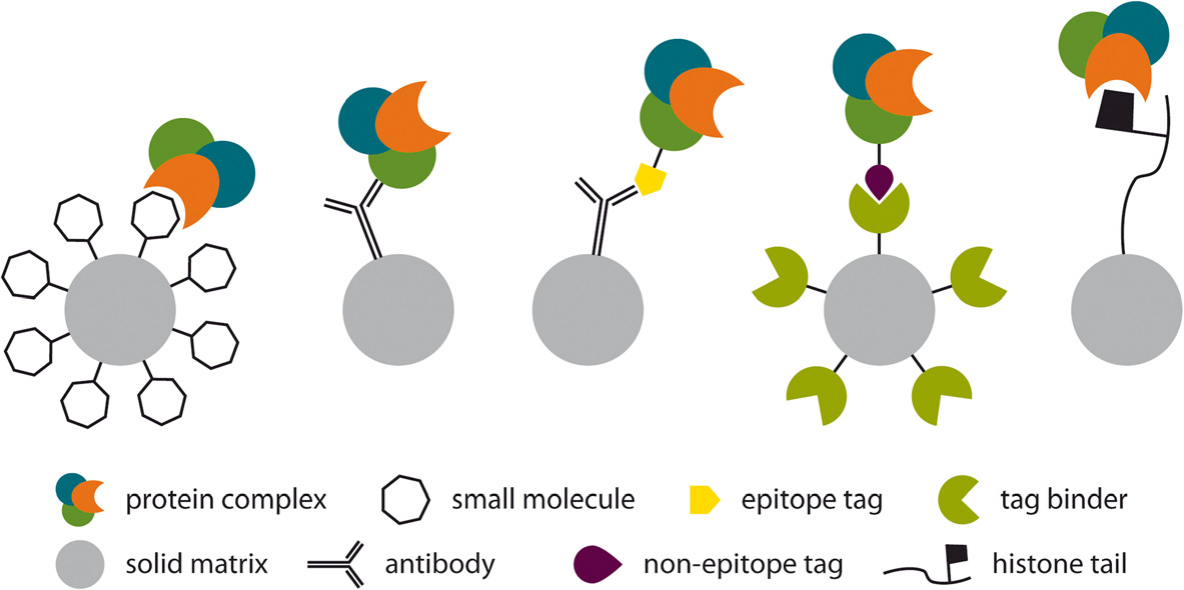
Strategies to capture histone-modifying complexes. These approaches include co-purifications via small molecule affinity probes, antibodies against native protein compounds, antibodies against epitope tags, non-epitope tag/tag binder couple, and bait peptides mimicking histone tails

Epigenetic modifications are carried out in a dynamic manner and are executed by multi-protein complexes, which can be described as writers that add posttranslational modifications (PTMs), erasers that remove them, and readers that provide PTM-dependent docking sites for additional functionally relevant protein complexes [59]. Even though many proteins involved in each of the tasks have been identified, epigenetic regulation occurs on the level of more complex protein interactions, which has gained rising interest during the past decade [60]. The study of protein complex composition has benefited from the combination of affinity purification (e.g., tandem affinity purification (TAP)) with mass spectrometry based proteomics [61]. Mass spectrometry has proven to be a useful tool not only for protein discovery but also to complement biochemistry based functional studies [62]. Hereinafter, we will describe proteomic strategies used to discover and characterize histone-modifying protein complexes. We will highlight the fact that in epigenetics, more than in other fields of research, it is protein complexes rather than single gene product enzymes that carry out the functions of writers, erasers, or readers. Because of this high level of complexity, typically several different proteomic methods need to be combined, in order to elucidate protein complex composition or the mechanism of action of drugs. These approaches include co-purifications via immobilized drugs, antibodies against native protein complex constituents, affinity matrices against epitope tags, or other recombinant tags and bait peptides (Fig. 2; Table 1).

**Table 1:** Studied histone-modifying complexes sorted by identification strategies

	Identification strategy				
Protein complex	Immobilized drug	Antibody against protein complex constituent	Antibody against epitope tags	Recombinant tags other than epitope taps	Bait (histone) tail peptide
Writer					
HAT			[82]	[99, 100]	
PMT	[56]		[84, 87]	[102, 105, 129]	[120]
Eraser					
HDAC	[20, 23, 29, 31, 63, 130]	[20, 70]	[88, 92, 109]	[131]	
Lysine demethylases	[52]		[85]	[71, 107]	[119]
Lysine decrotonylase					[117]
Reader					
BET	[63, 39]	[39]	[94]	[108]	[39]
Methyl-lysine- and/or methyl-arginine binding domain-containing protein				[76, 103, 110]	[76, 112, 113, 115]
PHD-containing proteins			[95]	[81, 101]	[116]
Phosphor-threonine/phosphor-serine binding protein	[111]				[111, 118]

*HAT* histone acetyl transferase, *PMT* protein methyl transferase, *HDAC* histone deacetylase, *BET* bromodomain and extraterminal domain family, *PHD* plant homodomain

### Chemoproteomics: pulldowns with immobilized drugs and other approaches

With the trapoxin matrix described earlier, Taunton et al. in 1996 were able to capture and identify not only HDAC1 for the first time but also RBBP4 (RbAp48) which was also lost from the matrix when trapoxin or trichostatin were set to compete for binding. Further experiments allowed them to indicate that HDAC1 was the enzymatic subunit while RBBP4 was serving a function of histone targeting [28]. Bantscheff et al. made use of their affinity matrix (vide infra) to also study the HDAC complex subunits that could be co-enriched and co-inhibited with free drugs. The authors characterized at least four different known HDAC complexes, including CoREST, NuRD, Sin3, and NCoR, scaffolded by ELM-SANT domain proteins as specific targets for subsets of 16 HDACi. In addition to the bead-immobilized drug, a competing inhibitor was added to the incubation mixture prior to affinity purification. Subsequent quantitative mass spectrometry-based analysis enabled the determination of HDAC complex specific dissociation constants for the various drugs. Bidirectional hierarchical clustering of dissociation constants against more than 500 proteins and the 16 HDACi suggested the existence of an additional unknown mitotic deacetylase complex (MiDAC). Moreover, the study showed a high selectivity of aminobenzamide for the HDAC3-NCor complex and little binding to the HDAC Sin3 complex [20]. In an extension of the chemical proteomics strategy, the matrix was used to evaluate the binding kinetics of hydroxamate and aminobenzamide inhibitors to the HDAC megadalton repressor complexes. Most remarkably, it was shown that, whereas hydroxamate inhibitors establish an equilibrium within second with the targets, it takes aminobenzamide inhibitors minutes for HDAC3/NcoR complex and hours for most of the complexes. Strikingly, it was also found that, for this scaffold, HDAC1/2 are not accessible when part of the Sin3 complex [63]. The Yates and Gottesfeld laboratories teamed up to use the pimelic diphenylamid probe described earlier for the study of HDAC complexes in Friedreich’s ataxia patient iPSC-derived stem cells. After their pulldowns, they simply compared the set of proteins enriched by the probe [31] to the interactome of all HDACs as defined by Cristea and coworkers in a study that we describe later in this review [64]. As mentioned earlier, Cravatt and coworkers synthesized a set of HDACi-BPyne. One of the goals was to change the position of the cross-linker, in order to reach proteins interacting with the HDACs the probes were binding to. SAHA-BPyne could itself enrich CoREST, MBD3, MTA1, MTA2, and p66b and the authors could hence compare the abundance of the complexes across a range of cell lines [23, 30]. Similarly, the group of Petukhov extended their range of HDAC probes featuring a 3-azido-5-azidomethylbenzyloxy moiety (which we described earlier for Vorinostat) and studied by western blot which of these “nanoruler” probes could cross-link SMRT while binding to HDAC3. They concluded that in the physiologically relevant conditions of HT29 cell lysates, SMRT is bound to HDAC3 in another conformation than in the X-ray structure [65].

This chemoproteomic approach is inherently limited by the availability of potent molecules targeting the protein complexes of interest as starting point to create linkable analogues. However, for many enzymes, no inhibitor is known or the synthesis of a suitable analogue appears too tedious. Antibodies can then be an alternative. Preferentially, they should be an orthogonal technique used in complement.

### Immunoaffinity purification of native protein complexes

Among antibody-based purifications of protein complexes, antibodies can target either native protein subunits or recombinant epitope tags. The advantage of antibodies targeting protein subunits is the ability to study physiologic complexes with a stoichiometry that is unaltered by overexpression [66–68].

For example, Liang et al. studied transcription-regulating complexes by capturing endogenous proteins. The transcription factors Nanog and Oct-4 are important for maintaining self-renewal and undifferentiated state in embryonic stem cells [69]. Immunoaffinity purifications (IAP) of Nanog and Oct4 from mouse embryonic stem cells revealed interactions with several repression complexes, including members of the HDAC chromatin remodeling complex NuRD and the repressor complex Sin3A. These interactions are relevant for embryonic cell fate decisions like self-renewal and pluripotency [70].

IAP when used in combination with chemoproteomics methods can help to deconvolute data from immobilized drug pulldowns: The latter often enrich mixtures of multiple protein complexes that are then co-eluted from the affinity matrix. For instance, immobilized pan-HDAC inhibitors affinity-capture all HDAC1-, HDAC2-, or HDAC3-containing protein complexes simultaneously. Therefore, proteomic analysis of all co-eluting proteins is not sufficient for unanimous characterization of individual protein complexes. In contrast, when chemoproteomics studies like these are complemented with IAPs using antibodies against known and unique subunits of single HDAC1, HDAC2, and HDAC3 complexes, e.g., antibodies against Sin3A, then comparison to proteins captured by immobilized HDAC inhibitors enables characterization of individual HDAC protein complexes [20]. Moreover, comparison of chemo- and IAP proteomics data can lead to the identification of novel HDAC protein complexes [20]. Finally, it enables the differentiation of direct HDACi binding targets from HDAC complex members [7].

A similar set of combined chemoproteomics, IAP proteomics, and additional affinity-capture proteomics experiments using compounds targeting the acetyl lysine recognizing bromodomain and extra terminal (BET) family of proteins and antibodies against individual BET complex subunits led to the elucidation of a potentially new therapeutic option for mixed lineage leukemia (MLL): Dawson et al. combined immobilized BET inhibitor pulldowns with IAPs and pulldowns using acetylated histone peptides and revealed association of the BET proteins BRD3 and BRD4 with the polymerase-associated factor complex (PAFc) and the super elongation complex (SEC), a key regulator of transcriptional elongation. Since translocation and fusion of the *mll* gene with members of the PAFc or SEC protein complexes has been implicated in leukemogenesis [71, 72], this newly discovered protein-protein interaction drew attention to the possible merit of using BET inhibitors for MLL leukemia treatment [39].

Despite the fact that antibodies raised against moieties of epigenetic proteins have proven their value, currently available literature suggests that these are rarely used. The main reasons may be the high cost and time requirement of raising antibodies and the frequently unsatisfying specificity that render them unsuitable for proteomics studies. Depending on the epitope that the antibody was raised against, antibody binding might also interfere with relevant protein-protein interactions [73].

### Antibody against epitope tags

Epitope tags are short peptide sequences attached to proteins during cloning and recombinant protein expression. They are frequently used for antibody-based detection or affinity (co-) purification purposes. The repertoire of common epitope tags, including FLAG, HA, and c-myc tags, has been reviewed elsewhere [74]. The main advantage of utilizing epitope tags for affinity purifications is the availability of very well characterized high quality anti-epitope tag antibodies.

For a long time, experimental strategies involving cellular expression of epitope or otherwise tagged proteins and their incorporation of tagged recombinant proteins into complexes in cell culture have been hampered by non-physiological and often poorly reproducible expression levels and, consequently, undefined stoichiometry. Many expression vectors that are typically used in molecular and cell biology, e.g., cytomegalovirus (CMV) promoters, are designed for high protein expression. Very high yield of the tagged bait protein per cell is, however, not desired in protein complex research, as many true interacting proteins are expressed at low endogenous levels and thus may be titrated out in the process. As a consequence, highly overexpressed bait proteins are often associated with few interacting proteins in low quantities that may evade identification. It has been recognized many years ago that alternative expression systems such as retroviral vectors that drive expression from long terminal repeat promoters may be beneficial and favor low bait protein expression consistent with more physiological protein complex formation [66, 75].

In a seminal 2010 study, Vermeulen et al. overcame this issue of difficult-to-control protein expression and protein complex stoichiometry by using a bacterial artificial chromosome (BAC) expression system [76]. BACs enable the gene expression from endogenous promoters in the presence of most regulatory genetic elements [77] or a knock-in of the recombinant protein [78]. They studied multiple trimethyl lysine reader protein complexes using a method called BAC transgeneOmics. Trimethyl lysine readers were initially captured using methylated prey peptides and identified using bottom-up proteomics. Promising candidates were then expressed as green fluorescent protein (GFP)-tagged proteins in HeLa cells. Protein complexes were extracted and purified in a single step using GFP-affinity resin. SILAC labeling enabled the reliable differentiation of specific binders from cell medium contaminants. For example, the study identified a double tudor domain in the C-terminus of Sgf29 as the H3K4me3-binding unit of the SAGA-complex. Moreover, they found a PWWP domain as putative H3K36me3 binding motive [76]. BAC-GFP transgenic HeLa cells have also been used to study the H3K4me3 reading EMSY complex. The latest quantitative mass spectrometry technology was used to analyze GFP-pulldowns of different EMSY-subunits. The transcription factor ZNF131 was identified, which recruits EMSY to active promoters [79].

In the field of epigenetics, epitope tagging has been the most widely used strategy for analysis of epigenetic protein complexes:

Proteins of the MYST family are highly conserved histone acetyl transferases (HATs) in eukaryotes, which typically possess a chromodomain and a zinc finger. One important member is the HAT HBO1 [80]. Purifications of MYST complexes revealed a tetrameric structure, which consists of a scaffold protein core, an ING tumor suppressor protein, and a catalytic enzyme unit [81]. Co-IAP FLAG-tagged BRPF1 as bait revealed the formation of novel MYST complexes, whose histone acetylation specificity depends on the scaffold protein core. HBO1 in a complex with JADE acetylates histone H4, whereas HBO1 in a complex with BRPF1 acetylates histone H3 [82].

The polycomb repressive complex 2 (PRC2) is one of the main histone methyl transferases inducing transcriptional silencing of chromatin. PRC2 consists of three core proteins: enhancer of zeste 2 (EZH2), embryonic ectoderm development (EED), and suppressor of zeste 12 homolog (SUZ12) [83]. Shen et al. found that *Ezh2* knockout did not abolish H3K27 methylation in mouse embryonic stem cells. Pulldowns of FLAG- and biotin-tagged EED from MESCs found the EZH2 homolog EZH1 in a non-canonical PRC2 complex. EZH1 preserves H3K27 methylation mark on chromatin of development-related genes in EZH2-deficient cells, which suggests that EZH1 safeguards the identity of embryonic stem cells [84]. IAP of FLAG-EZH2 from 293F cells revealed an interaction with Jarid2, a Jumonji C demethylase domain-containing protein that apparently lacks enzymatic activity. Jarid2 recruitment to a promoter triggered co-recruitment of PRC2 and increased methyltransferase activity [85].

Another histone methyl transferase complex is the MLL/COMPASS group of SET-domain histone methyltransferases (SET1/MLL). SET1/MLL and PRC2-complexes have been shown to often regulate the same promoter targets [86]. Van Nuland et al. performed a quantitative study on human SET1/MLL histone methyltransferase complexes. The expression of GFP-tagged subunits of COMPASS-like complexes gave insights to the stoichiometry of different SET1/MLL1 complexes. In addition, Bap18-GFP bacterial artificial chromosomes were used for the expression of a core component of the NURF chromatin remodeling complex. Bap18 pulldowns revealed that Dpy30 protein is not only a core COMPASS subunit but also a subunit of NURF [87].

In addition, class III HDAC complexes, i.e., sirtuin protein complexes, have been studied by co-IAP of epitope tagged proteins. Purification of the FLAG-tagged histone deacetylase SIRT1 found an interaction with the tumor suppressor DBC1 [88]. SIRT1 has been shown to deacetylate p53 to promote cell survival [89]. Inhibition of SIRT1 by DBC1 initiates p53-mediated apoptosis [88]. SIRT1-mediated deacetylation of p53 is stimulated by the transcription factor Oct4 to maintain pluripotency of ESCs [90]. Nanog, Sox2, and Oct4 are critical transcription factors to maintain self-renewal capacity in embryonic stem cells [91]. IAP of epitope tagged Oct4 and four of its binding transcriptions factors (Sall4, Esrrb, Dax1, and Tcfcp2l1) from mouse embryonic stem cells, in turn, revealed the complex interaction network of Oct4. The interaction of Oct4 and the nucleosome remodeling deacetylase (NuRD) HDAC complex member SALL4 stresses its role in modulating epigenetic silencing [92].

Joshi et al. gave a comprehensive overview on the interactors of the HDAC family. All human HDACs (HDAC 1–11) were GFP-tagged and co-immunopurified from CEM-T cells. Among the 200 novel identified interactions, a role of HDAC 11 in snRNP assembly and RNA processing was found and also the repertoire of known HDAC1 interacting proteins was expanded. Moreover, a metabolic labeling strategy was invented to study protein complex stabilities. For that, light-labeled GFP-HDAC expressing cells were mixed with heavy-labeled wild-type cells during co-immunopurification. The comparison of isotope protein ratios enabled conclusions on the exchange ratios of protein complex members. The approach showed that exchange ratios in HDAC1 containing complexes are rather slow and therefore more stable, whereas association of transcription factors is less stable [64].

The bromodomain protein Brd4 contains a pTEFb interaction domain. Binding of Brd4 to pTEFb stimulates transcriptional activation [93]. A co-purification of HA-tagged N-terminal domain of Brd4 revealed that the extraterminal domain of Brd4 recruits different effectors including NSD3 and JMJD6, which mediates transcriptional activation independent of pTEFb [94].

Mutations in methyl reader PHF6 are associated with the neurodegenerative disease Börjeson-Forssman-Lehmann syndrome as well as with MLL and T-ALL leukemias. PHF6 has been FLAG-tagged and immunoaffinity purified to identify interacting proteins. The experiment brought to light an interaction with the NuRD deacetylation complex and, hence, implicated PHF6 in chromatin regulation [95].

### Recombinant tags other than epitope tags

Aside from epitope tags, two other classes of protein tags for purification purposes have been extensively used: The first class consists of proteins (e.g., glutathione-S-transferase (GST)) or peptides like polyhistidine that bind to immobilized small molecules. The second class includes (poly)peptides such as calmodulin binding peptide or protein A that bind to immobilized proteins such as calmodulin and immunoglobulin, respectively [96]. In some cases two such affinity moieties have been combined in a single expression vector. Such tags have been developed especially for tandem affinity purification (TAP) mass spectrometry and are referred to as TAP tags. The rationale underlying the use of TAP tags is based on the notion that a single affinity purification step is often not sufficient to obtain pure protein (complexes). In contrast, TAP tags enable two-step affinity purifications under “native” conditions (i.e., pH 7.4 and physiological salt concentration) to reduce unspecific binders. Moreover, the design of TAP tags aims for low molecular weight (<20 kDa) to limit interference with protein folding [68]. The classical TAP tag consists of protein A and calmodulin binding peptide (CBP). The linker region between the two tags contains a rare protease recognition site, which enables cleaving the CBP-tagged protein off of the IgG-bound protein A by tobacco edge virus protease [97].

Doyon et al. used a TAP-tagging approach to study protein complexes containing inhibitor of growth (ING) proteins of the PHD domain-containing family that are involved in both tumor suppression and oncogenesis. They elucidated a central role of ING proteins in regulating chromatin acetylation, as several TAP-tagged ING-family members were found to associate with protein complexes involved in histone acetylation and deacetylation [81]. For example, ING2 associates with an HDAC complex, and its knockdown, has been shown to suppress cancer, thus identifying ING2 as a potential anti-cancer drug target [98]. ING4 and ING5 associate with HAT complexes [81].

The ING1 ortholog YNG1 is a member of the NuA3 histone acetylase complex in yeast. Different laboratories purified TAP-tagged subunits of the NuA3 complex to gain insights into its composition, regulation, and function [99–101]. Purification of TAP-tagged YNG1 found YNG1 to mediate NuA3 histone acetylation at H3K14. The pulldown also identified the previously uncharacterized PWWP domain protein Pdp3 as a member of the NuA3 complex [101]. Studies on the network of Sas3p, a HAT in the NuA3 complex, using a TAP-tag purification approach confirmed the association of Pdp3 with the NuA3 complex [100]. The use of TAP-tagged Pdp3 enabled the purification and classification of a NuA3b complex, which is functionally different from the non-Pdp3 containing NuA3a complex. This novel NuA3b complex mediates transcriptional elongation by binding to H3K36me3 in a Pdp3 regulated manner [99].

The histone methyl transferase SUV420H2 is a mark for pericentric chromatin. The Angrand laboratory TAP-tagged SUV420H2 and isolated its interaction partners from the nuclear extracts of HeLa cells. The group identified the heterochromatin protein HP1 as a major interaction partner of SUV420H2 [102]. In subsequent publications, the same laboratory studied members of the HP1 protein family, also referred to as chromobox homolog (CBX). HP1 isotypes were TAP-tagged and interacting partners tandem affinity purified. Those publications focused on the differences in protein complex composition among the HP1/CBX proteins [103, 104].

PRC1 is a histone methyl and ubiquitin transferase complex, which belongs with PRC2 to the family of polycomb group (PcG) complexes. TAP-based proteomics on PRC1 complex members found that the ubiquitin ligases RING1 A/B are members of all PRC1 complexes, whereas composition of the ring finger motif containing PCGF proteins varies. Six different PRC1 complexes were defined based on the contained PCGF protein. Moreover, incorporation of a RING1 binding protein, RYBP, prevents association of canonical complex members like CBX. Knock down of RYBP revealed its importance for embryonic stem cell proliferation [105]. The lysine specific demethylase 1 (LSD1) was the first discovered histone demethylase [106]. Its isoform LSD1+8a shows strong expression in neurons. Purification of TAP-tagged LSD1+8a from SH-SY5Y neuronal cell line revealed an interaction with the supervillin protein (SVIL). SVIL colocalizes with LSD+8a in promoter regions and functions as a cofactor for LSD+8a-mediated H3K9me2 demethylation [107]. The transcription factor Snail1 contains a SNAG domain, which shows structural similarity to histone H3 peptide. Purification of TAP-tagged Snail1 found an interaction with LSD1. The interaction of LSD1 with this molecular hook represses gene expression and results in decreased cell migration [71].

The bromodomain (BRD) protein ZMYND8 is a multidomain protein, which contains beside its BRD a plant homology (PHD) domain and a PWWP domain. Purification of SFB-tagged (S-tag, Flag epitope tag, and streptavidin-binding peptide tag) ZMYND8 found interactions with NuRD and BRAF HDAC complexes, which are important for promoting homologous recombination upon DNA damage [108].

Kloet et al. performed quantitative interaction studies on the NuRD HDAC complex. The group found that in contrast to the core units, binding to some zinc finger-containing proteins like SALL4 is salt-sensitive. Cross-linking MS was performed to identify intermolecular interactions between the methyl-CpG-binding protein MBD3 and the NuRD complex. Moreover, co-purifications of His-GFP-MBD3 revealed that the histone chaperones RBBP4 and RBBP7 dynamically interact with the MBD3-NuRD complex [109].

A novel NuRD-type chromatin remodeling complex was identified by Kolla et al. The chromodomain containing protein CHD4 and FOG1 are transcription factors in the NuRD complex. Pulldown experiments of GST-FOG1 identified a CHD4 homolog CHD5, which forms a similar NuRD complex. This novel chromatin remodeling complex might be important for normal cell development and tumor suppression [110].

### Histone tail peptides as baits

A further approach to capture epigenetic regulating protein complexes are exposed protein tails, which mimic the recognition site of protein domains. To study epigenetic regulators, the peptides of choice are histone tails containing different modifications like methyl or acetyl groups [39, 76, 111].

Vermeulen et al. compared the binding of histone readers to modified prey peptides using a SILAC approach. The affinity purification matrix used was immobilized N-terminal histone H3 tails showing different modifications. Extracts from cells supplied with light amino acids were exposed to a non-methylated peptide whereas extracts from heavy-labeled cells were exposed to a methylated peptide. The experiment identified TFIID as a H3K4me3 binding protein [112].

Li et al. also studied Histone H3 methyl-lysine binding proteins using a SILAC approach. Their histone H3 N-terminal tail probe contained additional chemical modifications including a click chemistry sensitive alkyne group for avidin tagging as well as a benzophenone group for UV cross-linking. The approach constitutes an interface between exposing prey peptides to protein complexes and chemical tagging of subunits of the complex. The heavy-labeled cellular extracts were exposed to a non-methylated histone tail, whereas heavy-labeled extracts were exposed to a trimethylated lysine residue. The identification of a new H3K4Me3 binding protein (MORC3) was demonstrated [113]. In a subsequent publication, it was shown that the use of diazirine instead of benzophenone as a cross-linking group yielded a more sensitive probe to capture H3K4Me3 binding proteins [114].

Zegerman et al. found that methylation at H3K4 prevents the NuRD complex from binding to the tail of H3. The group exposed N-terminal residues of histone H3 to nuclear extracts. The NuRD complex co-purified with unmodified histone tails but not with a peptide containing a trimethylated lysine 4 [115].

A similar approach was used to identify the PHD finger protein BPTF that specifically binds to H3K4Me3. BPTF is a subunit of the NURF complex. Therefore, the finding forms another link between histone modifications and chromatin remodeling [116].

Unstructured N-terminal tails of histone H3 have also been used to study protein complexes binding to rarely studied modifications like crotonylation and phosphorylation [111, 117, 118].

The Kapoor laboratory studied proteins binding to phosphorylation at H3T3, using a modified histone H3 tail peptide. The approach identified a phosphorylation-dependent protein-protein interaction with the phos-binding protein survivin, which only occurs during mitosis [111]. Kunowska et al. identified a novel complex binding the double H3K9me3/S10ph modification by exposing nuclear extracts to respective histone peptides. The complex integrates members of the chromatin remodeling FACT complex [118].

Bao et al. exposed nuclear extracts to histone H3 tail peptides, which contain a crotonylation mark at lysine 4. They found that several sirtuins (SIRT1, SIRT2, SIRT3), which belong to the HDAC family, possess decrotonylase activity [117].

Immobilized histones tail peptides have also been used to study binding efficacy of epigenetic complexes upon inhibitor treatment [39, 119, 120].

## Conclusions

There is no doubt that proteomics can and will help on every step of the epigenetic drug discovery effort in the years to come. Hence, the studies that have been reviewed here are to be seen as a mere commencement. We believe that they constitute the surface of what is possible and that many discoveries using proteomics approaches lie ahead of us. Exciting developments should also settle or find their way in the epigenetics field, expanding the proteomics toolbox to study drug action and protein complex composition. For instance, the possibility to photo-cross-link inhibitors to beads without the need to synthesize linkable analogues is a promising approach [121] which could render pulldowns a routine characterization tool after any phenotypic screen. Another expansion of the toolbox is an approach based on the successful combination of cellular thermal shift assays (CETSA) and bottom-up proteomics [122, 123]. This innovation, also referred to as thermal proteome profiling (TPP), makes use of (small molecule) ligand-induced thermal stabilization of target proteins. When incorporated into quantitative proteomics workflows, it can be used for identification of direct and indirect drug targets in living cells, as was recently demonstrated using the HDACi panobinostat as a point in case [124]. Recent evidence also suggests that the study of epigenetic complexes and their inhibitors by proteomics is not the prerogative of tandem liquid chromatography mass spectrometry but that matrix-assisted laser desorption/ionization (MALDI) mass spectrometry may, especially when used as an imaging modality, play its part, as demonstrated by Munteanu et al.: they presented a cellular assay based on whole cell MALDI MS fingerprinting that enables label-free measurement of cellular potency and monitoring of drug target engagement of HDACi (panobinostat) without requiring any enrichment or sophisticated sample preparation steps [125, 126]. Moreover, MALDI MS imaging can visualize histone modifications and consequently drug action of histone-modifying small molecules in a spatially resolved manner, thereby opening up the door for a fruitful marriage of “omics” and imaging [125, 127]. Finally, as the boundaries of the epigenetics target space remains fuzzy with a complexity beyond the currently pursued drug discovery efforts [128], proteomics has a major role to play. With relatively unbiased methods, mass spectrometry-based proteomics indeed offer new opportunities to identify and characterize new epigenetics target/inhibitor couples which should find a much needed translation in the clinic.
